# Development of a Novel Tool for the Retrieval and Analysis of Hormone Receptor Expression Characteristics in Metastatic Breast Cancer via Data Mining on Pathology Reports

**DOI:** 10.1155/2020/2654815

**Published:** 2020-05-23

**Authors:** Kai-Po Chang, John Wang, Chi-Chang Chang, Yen-Wei Chu

**Affiliations:** ^1^Ph.D. Program in Medical Biotechnology, National Chung Hsing University, Taichung 402, Taiwan; ^2^Department of Pathology, China Medical University Hospital, Taichung 404, Taiwan; ^3^School of Medical Informatics, Chung-Shan Medical University, Taichung 402, Taiwan; ^4^IT Office, Chung-Shan Medical University Hospital, Taichung 402, Taiwan; ^5^Institute of Genomics and Bioinformatics, National Chung Hsing University, 145 Xingda Rd., South Dist., Taichung City 402, Taiwan; ^6^Institute of Molecular Biology, National Chung Hsing University, 145 Xingda Rd., South Dist., Taichung City 402, Taiwan; ^7^Agricultural Biotechnology Center, National Chung Hsing University, 145 Xingda Rd., South Dist., Taichung City 402, Taiwan; ^8^Biotechnology Center, National Chung Hsing University, 145 Xingda Rd., South Dist., Taichung City 402, Taiwan; ^9^Ph.D. Program in Translational Medicine, National Chung Hsing University, 145 Xingda Rd., South Dist., Taichung City 402, Taiwan; ^10^Rong Hsing Research Center for Translational Medicine, National Chung Hsing University, 145 Xingda Rd., South Dist., Taichung City 402, Taiwan

## Abstract

Information about the expression status of hormone receptors such as estrogen receptor (ER), progesterone receptor (PR), and Her-2 is crucial in the management and prognosis of breast cancer. Therefore, the retrieval and analysis of hormone receptor expression characteristics in metastatic breast cancer may be valuable in breast cancer study. Herein, we report a text mining tool based on word/phrase matching that retrieves hormone receptor expression data of regional or distant metastatic breast cancer from pathology reports. It was tested on pathology reports at the China Medical University Hospital from 2013 to 2018. The tool showed specificities of 91.6% and 63.3% for the detection of regional lymph node metastasis and distant metastasis, respectively. Sensitivity in immunohistochemical study result extraction in these cases was 98.6% for distant metastasis and 78.3% for regional lymph node metastasis. Statistical analysis on these retrieved data showed significant difference s in PR and Her-2 expressions between regional and metastatic breast cancer, which is compatible with previous studies. In conclusion, our study shows that metastatic breast cancer hormone receptor expression characteristics can be retrieved by text mining. The algorithm designed in this study may be useful in future studies about text mining in pathology reports.

## 1. Introduction

Breast cancer is the second most lethal cancer worldwide, accounting for 626,679 deaths in 2018 [1]. These fatalities are primarily due to its potential to metastasize, with 28.8% of patients experiencing axillary lymph node metastases [2] and 20-30% of patients experiencing subsequent distant metastasis even if the cancer is found in an early stage [3]. Therefore, a study on the behavior of metastatic breast cancer is of particular importance in breast cancer treatment and public health. During the previous two decades of medical advancement, numerous novel molecular targets, such as LIFR [4], PI3K [5], and aldehyde dehydrogenase-1 [6], have been studied for prognosis prediction and target therapy for metastatic breast cancer, but none of them have been proven to be more valuable than the long-standing markers estrogen receptor (ER), progesterone receptor (PR), and human epidermal growth factor receptor 2 (erbb-2 or Her-2).

According to recent studies, molecular subtypes luminal A, luminal B, Her-2, and triple-negative, which are determined by these markers, are still relevant to the treatment and prognosis of metastatic breast cancer [7–10].

As important markers of special value, ER, PR, and Her-2 expression are routinely examined by immunohistochemical study [11–13] on all invasive breast cancer slides and are documented in pathology reports. Combined with the fact that occurrences of lymph node or distant metastatic breast cancer are frequently sampled for pathologic examination [14], a pathology report database may be an important resource for the hormone receptor expression status of metastatic breast cancer. However, extraction of these data can be a tedious task. Unlike surgical pathology reports for primary breast cancer, in which pathologists are required to report in certain forms [15] or a synaptic report system [16–18], there are no required forms for reporting metastatic carcinoma in most institutions, and most of these reports stay in free text form. Retrieving these data requires text mining approaches to avoid tedious manual work. As we have discussed in a previous article [19], most general medical text mining utilities do not process immunohistochemical study results [20, 21], while those that do process immunohistochemical data use advanced natural language processing (NLP) methods [22, 23] and therefore will not be available in general hospital information system (HIS).

This difficulty can be solved by using simpler methods such as word/phrase matching, concept-match scrubbing [24], and semantic grammar-based concept finding [25] with clinical knowledge. We have shown in a previous publication [19] that regular expression-based word/phrase matching can be used to mine hormone receptor data for primary and recurrent breast cancer. In this article, we show that the text mining algorithm described in the previous publication can also be applied to metastatic breast cancer.

## 2. Materials and Methods

### 2.1. Data Retrieval and Preprocessing

**Figure 1:**
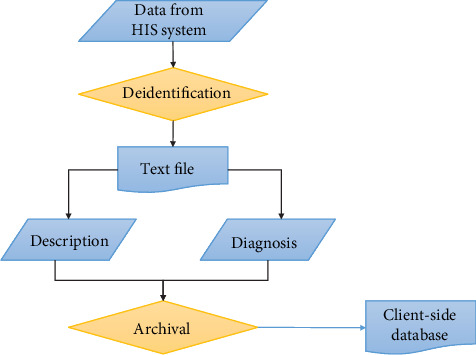
Data retrieval and preprocessing steps.

All pathology reports issued at the China Medical University Hospital (CMUH) from the years 2013 to 2018, estimated 200,000 reports, were first exported into pure text form. The patient data within the text file was then automatically deidentified using the method described by Neamatullah et al. [26] to eliminate violation of privacy and ethical concerns. A Python script [27] was designed to extract the pathology diagnosis and description columns from the text files and build a client-side database using SQLite3 [28]. The data retrieval and preprocessing steps are shown in Figure 1.

### 2.2. Retrieval of Metastatic Breast Cancer Cases

The authors first manually reviewed 50 pathology reports documenting regional lymph node metastatic breast cancer and 50 pathology reports documenting distant metastatic breast cancer. From these reports, it was seen that most pathology reports documenting a metastatic carcinoma had either “carcinoma, metastatic” or “carcinoma, involved” in the diagnosis. Those of breast origin were described as “breast origin” or “breast primary”. Regional lymph node metastatic tumors were described as “soft tissue, axillary” or “lymph node, axillary”, while distant metastatic tumor were described in the pattern “(any organ name other than axillary tissue), (procedure), carcinoma, metastatic/involved, and breast origin”.

**Figure 2:**
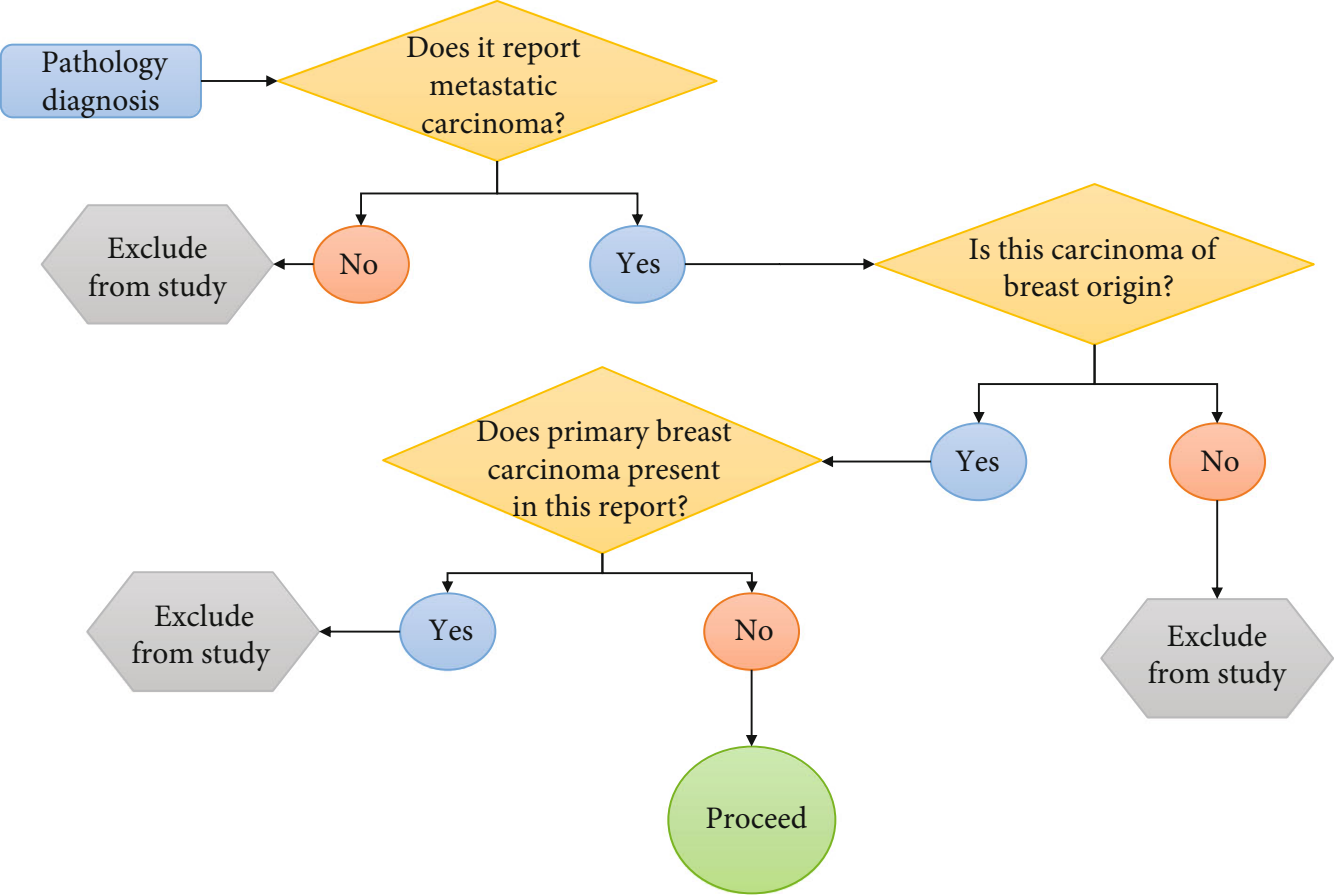
Protocol for searching metastatic breast cancer cases.

Based on these results, we designed our metastatic breast cancer finding algorithm according to the following strategy:

1. Each line from the diagnostic column is matched with the phrase “carcinoma, metastatic,” “carcinoma, involved,” or any phrase indicating metastatic carcinoma by a regular expression engine. If any of the lines matched one of the patterns, the report is passed to the next step for further processing
2. When one of the lines in the diagnosis indicates metastatic carcinoma, that line is checked for the presence of phrases that indicate breast origin, such as “breast primary” or “breast origin”. Any reports that show a match in these phrases is passed into the next step for examination
3. For reports that show evidence of metastatic carcinoma of breast origin, the whole diagnostic column is checked for the presence of signs of primary breast cancer. If any of the lines from the diagnostic column shows any phrase that represents primary breast cancer, the report is excluded from further analysis
4. Metastatic sites are parsed and recorded by another regular expression engine. 490 reports documenting metastatic disease (359 regional metastases, 131 distant metastases) are retrieved in this step. The search protocol is shown in Figure 2

### 2.3. Identification of Paragraphs Containing Immunohistochemical Study Results

A two-step regular expression matching engine for immunohistochemical study extraction, as described in our previous study on extracting immunohistochemical result of primary and recurrent breast cancer [19], was utilized. In the first step, the program attempted to match common forms in which pathologists express immunohistochemical study results.

**Figure 3:**
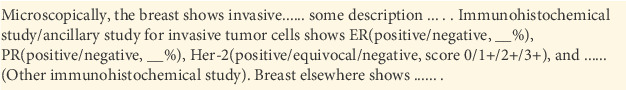
Reporting immunohistochemical study results as a sentence in the microscopic description.

**Figure 4:**
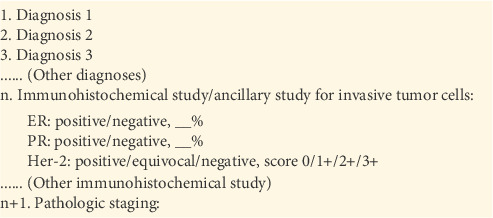
Reporting immunohistochemical study results as a solitary paragraph with multiple rows.

**Figure 5:**
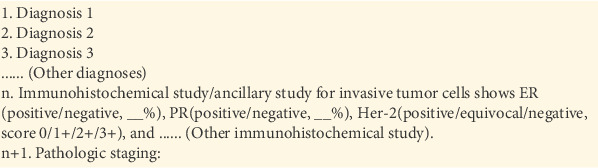
Reporting immunohistochemical study results as a solitary paragraph, with different studies separated by commas.

There is, however, a significant difference between identification of immunohistochemical study in primary/recurrent breast cancer and metastatic breast cancer. When reporting metastatic carcinoma, pathologists in our institution usually document immunohistochemical study results in the description rather than the diagnostic column; therefore, searching immunohistochemistry-containing paragraphs in the current study only involved parsing the description column (Figure 3) but not the diagnosis column (Figures 4 and 5). This approach can optimize the searching process without sacrificing sensitivity.

Paragraphs extracted from this step will then undergo the following steps for immunohistochemical study result extraction.

### 2.4. Extraction of Immunohistochemical Study Results

In institutes that are routinely accredited by the College of American Pathologists (CAP), such as our institute, the reporting format of ER, PR, and Her-2 result is regulated by guidelines [29, 30]. Therefore, in our method, the results of ER, PR, and Her-2 result are matched and extracted according to those guidelines. Since our laboratory applied the new 2018 CAP recommendations in 2019, so the ER, PR, and Her-2 results included in this study were issued using 2013 recommendation.

For ER and PR, positivity is required. If the result is positive, the expression percentage should be reported. Therefore, there would be three patterns: “ER/PR (positive, %)”, “ER/PR: positive, %”, and “ER (positive)”.

For Her-2 results, both positivity (positive, equivocal, and negative) and score (0, 1+, 2+, and 3+) are required. Therefore, there would be two patterns: “Her-2/Her2/HER2/HER-2 (positive/equivocal/negative, 0/1+/2+/3+, or score 0/1/2/3)” and “Her-2/Her2/HER2/HER-2: positive/equivocal/negative, 0/1+/2+/3+, or score 0/1/2/3, weak/moderate/strong staining in %”.

### 2.5. Recording of Results

The results are exported into a csv file by the program, recording each case in the form: “case ID, metastatic site, ER result, PR result, and Her-2 result”. If there is a failed extraction, the result is recorded as “None”.

### 2.6. Validation of Results

All cases and immunohistochemical study results were reviewed by two board-certificated pathologists (Kai-Po Chang and John Wang) for validation.

### 2.7. Statistical Analysis

For comparison of hormone receptor results between different metastatic sites, Pearson's Chi-squared test with Yates' continuity correction was done with the MASS package of R version 3.5.1 under Windows 10.

## 3. Results

### 3.1. Detection of Metastatic Breast Cancer Cases

Our program labeled 131 pathology reports as describing distant metastatic breast cancer, of which 83 were correctly labeled, resulting in a specificity of 63.3%. There were 359 pathology reports labeled as describing regional lymph node metastatic breast cancer, of which 329 were correctly labeled, resulting in a specificity of 91.6%.

Sensitivity could not be determined, since there is no cancer registry data for metastatic carcinoma. The results are summarized in Table 1.

Among the 83 cases of distant metastatic cancer, the metastatic sites include the nonregional lymph node (22 cases), bone (20 cases), brain (12 cases), liver (8 cases), gastrointestinal tract (8 cases), lung (7 cases), uterus (1 case), pleura (1 case), pelvic cavity (1 case), ovary (1 case), mediastinum (1 case), and urinary bladder (1 case). The results are summarized in Table 2.

### 3.2. Immunohistochemical Study Result Detection and Extraction

In the 83 cases documenting distant metastatic disease, the program detected immunohistochemical study results in 65 cases, with an error in documentation of the immunohistochemical study result in 1 case, resulting in a sensitivity of 78.3% and a specificity of 98.4%. In 329 cases documenting regional lymph node metastatic diseases, the program correctly detected immunohistochemical study results in 316 cases, resulting in a sensitivity of 98.1% and a specificity of 100%. The results are documented in Table 3.

Among the 64 cases of distant metastatic cases with correctly detected immunohistochemical study results, all were tested for ER, 52 were tested for PR, and 58 were tested for Her-2. Of the cases tested for ER, 36 (62.0%) were positive, and 28 (38.0%) were negative. Of the cases tested for PR, 12 (23.0%) were positive, and 40 (67.0%) were negative. Of the cases tested for Her-2, 23 (39.6%) were positive (score 3+), 11 (19.0%) were equivocal (score 2+), and 24 (41.4%) were negative (score 1+ or 0). The results are shown in Table 4.

Among the 322 cases of regional lymph node metastatic cases with correctly detected immunohistochemical study results, 308 were tested for ER, 91 were tested for PR, and 303 were tested for Her-2. Of the cases tested for ER, 198 were positive, and 110 were negative. Of the cases tested for PR, 52 were positive, and 29 were negative. Of the cases tested for Her-2, 103 were positive (score 3+), 95 were equivocal (score 2+), and 112 were negative (score 1+ or 0). The results are shown in Table 5.

### 3.3. Comparison of Hormone Receptor Expression between Lymph Node Metastatic Breast Cancers

After applying chi-squared tests to the above results, it was concluded that distant metastatic tumors had a significantly higher probability to be Her-2-positive and PR-negative than did regional metastatic tumors, while there was no significant difference between ER expression in regional and distant metastatic diseases. For details, please see Tables 6–8.

Our observation that distant metastatic tumors are more prone to be Her-2 positive and PR-negative may be consistent with previous studies that Her-2 positive and PR-negative tumor have higher incidence of distant metastasis.

### 3.4. Comparison of Hormone Receptor Expression between Major Metastatic Sites

According to our data, compared with bone and brain metastatic diseases, lung metastatic disease has a tendency to be more ER-positive and Her-2 positive, which is consistent with previous studies [31, 32]. However, there is no statistically significant difference in the chi-squared analysis, which is probably due to a low sample number. Details are shown in Tables 9–11.

## 4. Discussion

### 4.1. Specificity Issue of Distant Metastatic Case Detection

The most significant flaw in our approach on metastatic breast cancer mining is its low specificity in distant metastatic cases. Of the 47 cases in which the program marked the report as a metastatic carcinoma but it actually was not, most (35) of them were documenting soft tissue or skin of the chest wall involved in recurrent breast cancer, in which the case should have been labeled as recurrent disease, not metastatic disease. Of the remaining wrongly marked cases, 11 of the 12 were due to a particular special habit of some pathologists when reporting negative sentinel lymph nodes, in which a phrase “s/p breast cancer” is inserted to the diagnosis to specify that the patient has undergone previous surgery for breast cancer. The last case is an endometrial curettage report, in which the pathologist noted in the diagnosis that the patient was under tamoxifen treatment for breast cancer.

Chest wall recurrent cases misinterpreted as metastatic carcinoma occurred most often, but they may be the most easily handled. In our previous publication [19], we developed an algorithm that detects recurrent carcinoma at either the breast or chest wall. If combined with that algorithm, chest wall recurrent cases can be easily filtered out. The cases in which the pathologist mentioned breast cancer in otherwise nonmalignant reports is a more difficult issue, since interpretation of that phrase will require semantic understanding of the pathology report.

To solve this problem, rule-based approaches, such as one described by Hur et al. [33] for mining biomedical literature and another described by Yang et al. [34] for mining hospital records, may be developed. However, since the pathology reports are written quite liberally, it is questionable whether specific rules can be built to fit theoretically infinite numbers of possible writing combinations on a pathology report. A more recent text-mining method is distributional semantic modeling [35]. In this method, corpora of text are first given, and the relationships between all words, including similarity and relatedness, are measured by vector-assisted analysis of coexistence in the corpus. This approach maybe more feasible, since this method would recognize the semantics of pathology reports. Subgraph mining that deconstructs the whole pathology report into higher order elements (subgraphs) [36] may be helpful as well. With recent advancements in text mining technology, new methods will emerge, and the problem encountered in our study may be overcome.

### 4.2. Further Research Directions

This study confirmed the concern in our previous publication that a nonstandardized pathology report may pose a difficulty in text mining, but we have discussed in the previous paragraph that it can be solved. By altering regular expression patterns, multiple forms of pathology report writing can be parsed and mined. Another issue mentioned in our previous publication, variation in reporting immunochemical study result, is nevertheless still not solved. Since we only have reports from one institution, it is unknown if our program works in pathology reports elsewhere. Therefore, for researchers in text mining, exploring the various forms in which hormone receptors such as ER, PR, and Her-2 are expressed may be an interesting and realistic research target. As we have stated above, the detection of metastatic disease, because of its difficulty, is also a potential research project.

## 5. Conclusions

In conclusion, our program showed that in metastatic breast cancer, the ER, PR, and Her-2 immunohistochemical study data can be mined using simple word/phrase matching assisted by regular expression. The algorithm designed in this study may be useful in future studies about text mining in pathology reports.

## Figures and Tables

**Table 1:** Summary of the results of metastatic breast cancer detection.

Metastatic site	Cases labeled as metastatic carcinoma	Label correct	Specificity
Regional	359	329	91.6%
Distant	131	83	63.3%

**Table 2:** Summary of metastatic sites.

Metastatic site	Case number
Nonregional lymph node	22
Bone	20
Brain	12
Liver	8
GI tract	8
Lung	7
Others	6

**Table 3:** Summary of results of the extraction of immunohistochemical study result data.

Metastatic site	Case number	Result detected	Result correct	Sensitivity	Specificity
Regional	83	65	64	78.3%	98.4%
Distant	329	322	322	98.6%	100%

**Table 4:** Summary of immunohistochemical study results of distant metastatic tumors.

Marker	Positive	Equivocal	Negative	Not tested
ER	36 (62.0%)		28 (38.0%)	0
PR	12 (23.0%)		40 (67.0%)	12
Her-2	23 (39.6%)	11 (19.0%)	24 (41.4%)	8

**Table 5:** Summary of immunohistochemical study results of regional metastatic tumors.

Marker	Positive	Equivocal	Negative	Not tested
ER	198 (64.3%)		110 (35.7%)	14
PR	52 (57.1%)		29 (42.9%)	231
Her-2	103 (34.0%)	95 (31.3%)	112 (37.0%)	8

**Table 6:** Difference of ER expression between distant and regionally metastatic breast cancers.

ER result	Distant metastasis	Regional metastasis
Positive	36	198
Negative	28	110

*χ* ^2^ = 1.1422, df = 1, *p* = 0.2852.

**Table 7:** Difference of PR expression between distant and regionally metastatic breast cancers.

PR result	Distant metastasis	Regional metastasis
Positive	12	52
Negative	40	29

*χ* ^2^ = 19.835, df = 1, *p* = 8.444*e* − 06.

**Table 8:** Difference of Her-2 expression between expression between distant and regionally metastatic breast cancers.

Her-2 result	Distant metastasis	Regional metastasis
Positive	23	103
Equivocal	11	95
Negative	24	112

*χ* ^2^ = 37.556, df = 2, *p* = 6.995*e* − 09.

**Table 9:** ER expression status of major metastatic sites.

ER result	Bone	Liver	Lung
Positive	8	4	1
Negative	4	4	1

*χ* ^2^ = 3.5011, *df* = 2, *p* = 0.1737.

**Table 10:** PR expression status of major metastatic sites.

PR result	Bone	Liver	Lung
Positive	4	2	1
Negative	7	5	5

*χ* ^2^ = 4.6286, *df* = 2, *p* = 0.09884.

**Table 11:** Her-2 expression status of major metastatic sites.

Her-2 result	Bone	Liver	Lung
Positive	3	3	6
Equivocal	3	0	5
Negative	4	1	0

*χ* ^2^ = 7.5455, df = 4, *p* = 0.1097.

## Data Availability

The data generated by the script is available as supplementary data S1. Sample code was deposited in GitHub https://github.com/medchem/breastmeta/.

## Supplementary Materials

Supplementary Materials Supplementary data 1: original research data of breast cancer cased analyzed by the script.

## References

[1] Bray F., Ferlay J., Soerjomataram I., Siegel R. L., Torre L. A., Jemal A. (2018). Global cancer statistics 2018: GLOBOCAN estimates of incidence and mortality worldwide for 36 cancers in 185 countries. *CA: A Cancer Journal for Clinicians*.

[2] Lee J. H., Kim S. H., Suh Y. J., Shim B. Y., Kim H. K. (2010). Predictors of axillary lymph node metastases (ALNM) in a Korean population with T1-2 breast carcinoma: triple negative breast cancer has a high incidence of ALNM irrespective of the tumor size. *Cancer Research and Treatment*.

[3] Abe O. (2005). Effects of chemotherapy and hormonal therapy for early breast cancer on recurrence and 15-year survival: an overview of the randomised trials. *Lancet*.

[4] Chen D., Sun Y., Wei Y. (2012). LIFR is a breast cancer metastasis suppressor upstream of the Hippo-YAP pathway and a prognostic marker. *Nature Medicine*.

[5] Gonzalez-Angulo A. M., Ferrer-Lozano J., Stemke-Hale K. (2011). PI3K pathway mutations and PTEN levels in primary and metastatic breast cancer. *Molecular Cancer Therapeutics*.

[6] Charafe-Jauffret E., Ginestier C., Iovino F. (2010). Aldehyde dehydrogenase 1-positive cancer stem cells mediate metastasis and poor clinical outcome in inflammatory breast cancer. *Clinical Cancer Research*.

[7] Telli M. L. (2016). Triple-negative breast cancer. *Molecular Pathology of Breast Cancer*.

[8] Akrami M., Tahmasebi S., Zangouri V., Hosseini S., Talei A. R. (2017). Metastatic behavior of breast cancer subtypes. *Multidisciplinary Cancer Investigation*.

[9] Tran B., Bedard P. L. (2011). Luminal-B breast cancer and novel therapeutic targets. *Breast Cancer Research*.

[10] Malik F., Ithimakin S., Day K. (2012). Abstract 3470: HER2 drives luminal breast cancer stem cells in the absence of HER2 amplification: implications for efficacy of adjuvant trastuzumab. *Cancer Research*.

[11] Nadji M., Gomez-Fernandez C., Ganjei-Azar P., Morales A. R. (2005). Immunohistochemistry of estrogen and progesterone receptors Reconsidered. *American Journal of Clinical Pathology*.

[12] Harvey J. M., Clark G. M., Osborne C. K., Allred D. C. (1999). Estrogen receptor status by immunohistochemistry is superior to the ligand-binding assay for predicting response to adjuvant endocrine therapy in breast cancer. *Journal of Clinical Oncology*.

[13] Carlson R. W., Moench S. J., Hammond M. E. H. (2006). HER2 testing in breast cancer: NCCN Task Force report and recommendations. *Journal of the National Comprehensive Cancer Network*.

[14] Tsao M. N., Rades D., Wirth A. (2012). Radiotherapeutic and surgical management for newly diagnosed brain metastasis(es): An American Society for Radiation Oncology evidence-based guideline. *Practical Radiation Oncology*.

[15] Tobias J., Chilukuri R., Komatsoulis G. A. (2006). The CAP cancer protocols – a case study of caCORE based data standards implementation to integrate with the Cancer Biomedical Informatics Grid. *BMC Medical Informatics and Decision Making*.

[16] Casati B., Haugland H. K., Barstad G. M. J., Bjugn R. (2014). Implementation and use of electronic synoptic cancer reporting: an explorative case study of six Norwegian pathology laboratories. *Implementation Science*.

[17] Leong A. S.-Y. (2001). Synoptic/checklist reporting of breast biopsies: has the time come?. *The Breast Journal*.

[18] Srigley J. R., McGowan T., MacLean A. (2009). Standardized synoptic cancer pathology reporting: a population-based approach. *Journal of Surgical Oncology*.

[19] Chang K. P., Chu Y. W., Wang J. (2019). Analysis of hormone receptor status in primary and recurrent breast cancer via data mining pathology reports. *Open Medicine*.

[20] Savova G. K., Masanz J. J., Ogren P. V. (2010). Mayo clinical Text Analysis and Knowledge Extraction System (cTAKES): architecture, component evaluation and applications. *Journal of the American Medical Informatics Association*.

[21] Aronson A. R. Effective mapping of biomedical text to the UMLS Metathesaurus: the MetaMap program.

[22] Luo Y. (2014). *Towards unified biomedical modeling with subgraph mining and factorization algorithms*.

[23] Luo Y., Sohani A. R., Hochberg E. P., Szolovits P. (2014). Automatic lymphoma classification with sentence subgraph mining from pathology reports. *Journal of the American Medical Informatics Association*.

[24] Berman J. J. (2003). Concept-match medical data scrubbing: how pathology text can be used in research. *Archives of Pathology & Laboratory Medicine*.

[25] Nassif H., Woods R., Burnside E., Ayvaci M., Shavlik J., Page D. Information extraction for clinical data mining: a mammography case study.

[26] Neamatullah I. (2008). Automated de-identification of free-text medical records. *BMC Medical Informatics and Decision Making*.

[27] Rossum G. V. (2003). The Python Language Reference Manual, Release 3.5.1. *Network Theory*.

[28] Owens M. (2006). The definitive guide to SQLite. https://www.google.com/books?hl=zh-TW&lr=&id=VsZ5bUh0XAkC&oi=fnd&pg=PR17&dq=sqlite&ots=u77Ngm44A6&sig=w46spF6bGTnbgPwuEbfrlvxUBhY.

[29] Fitzgibbons P. L., Murphy D. A., Hammond M. E., Allred D. C., Valenstein P. N. (2010). Recommendations for validating estrogen and progesterone receptor immunohistochemistry assays. *Archives of Pathology & Laboratory Medicine*.

[30] Wolff A. C., Hammond M. E., Hicks D. G. (2013). Recommendations for human epidermal growth factor receptor 2 testing in breast cancer: American Society of Clinical Oncology/College of American Pathologists clinical practice guideline update. *Journal of Clinical Oncology*.

[31] Nguyen P. L., Taghian A. G., Katz M. S. (2008). Breast cancer subtype approximated by estrogen receptor, progesterone receptor, and HER-2 is associated with local and distant recurrence after breast-conserving therapy. *Journal of Clinical Oncology*.

[32] Savci-Heijink C. D., Halfwerk H., Hooijer G. K. J., Horlings H. M., Wesseling J., van de Vijver M. J. (2015). Retrospective analysis of metastatic behaviour of breast cancer subtypes. *Breast Cancer Research and Treatment*.

[33] Hur J., Schuyler A. D., States D. J., Feldman E. L. (2009). SciMiner: Web-based literature mining tool for target identification and functional enrichment analysis. *Bioinformatics*.

[34] Yang H., Spasic I., Keane J. A., Nenadic G. (2009). A text mining approach to the prediction of disease status from clinical discharge summaries. *Journal of the American Medical Informatics Association*.

[35] Marelli M., Bentivogli L., BaroniM B. R., Menini S., Zamparelli R. SemEval-2014 Task 1: evaluation of compositional distributional semantic models on full sentences through semantic relatedness and textual entailment. http://clic.cimec.unitn.it/marco/publications/marelli-etal-semeval14-task1.pdf.

[36] Luo Y., Xin Y., Hochberg E., Joshi R., Uzuner O., Szolovits P. (2015). Subgraph augmented non-negative tensor factorization (SANTF) for modeling clinical narrative text. *Journal of the American Medical Informatics Association*.

